# Investigating the Impact of Different Drying Methods on the Aroma of Citri Reticulatae Pericarpium Using GC–MS/GC–IMS and E-Nose Technology

**DOI:** 10.3390/foods15071117

**Published:** 2026-03-24

**Authors:** Aygul Alim, Chengfei Huang, Xin Zhao, Saren Gaowa, Runrong Zhang, Junrong Zhang, Xueqing Zhang, Yuanbao Jin, Wenzhong Hu

**Affiliations:** School of Life Science, Zhuhai College of Science and Technology, No. 8 Anji East St., Jinwan Dist, Zhuhai 519041, China; aygulalim@sina.com (A.A.); ysxy@zcst.edu.cn (C.H.); 23383@zcst.edu.cn (X.Z.); kuailexiaosa@sina.com (S.G.); zrr163@126.com (R.Z.); zhangjunrong@zcst.edu.cn (J.Z.); 17666276137@163.com (X.Z.); jinyuanbao@zcst.edu.cn (Y.J.)

**Keywords:** volatile compounds, sensory evaluation, multivariate analysis, aroma profile, drying

## Abstract

Drying and storage conditions play a critical role in shaping the quality of aged citrus peel. This study investigated the effects of different processing strategies on the volatile composition, microstructure, and sensory characteristics of five-year-aged Citrus Reticulata ‘Chachi’ Peel (CRP). Four treatments were evaluated using SPME/GC–MS, GC–IMS, electronic nose analysis, sensory assessment, scanning electron microscopy (SEM), and multivariate statistical tools. GC–IMS identified 96 volatile compounds, demonstrating that aging combined with varied drying–storage conditions promoted the formation of diverse aroma-active substances. Terpenes and related compounds predominated, with the indoor-dried and warehouse-stored XH sample showing significantly higher concentrations of key terpenoids and sesquiterpenes, including α-terpineol, γ-muurolene, germacrene, β-selinenol, α-farnesene, and nerolidol. These compounds contributed to enhanced citrus, floral, fruity, and woody notes. Principal component analysis of electronic nose data (93.46% cumulative variance) clearly distinguished XH from other samples. Sensory results supported instrumental findings, indicating stronger fruity and sweet attributes in XH and C, while sun-dried samples exhibited more hay-like characteristics. SEM revealed better structural integrity in indoor-dried samples, potentially facilitating volatile retention. Overall, indoor drying and controlled storage improved aroma complexity and sensory quality, providing a scientific basis for optimized CRP processing.

## 1. Introduction

Citrus Reticulata Peel (CRP) is extensively produced in Guangdong, Fujian, Sichuan, Chongqing, and Zhejiang provinces in southern China [1]. As a well-recognized traditional Chinese medicinal herb, the quality of CRP is enhanced with prolonged aging, which is attributed to the temporal variations in its volatile oil components [2]. CRP contains over 300 volatiles (terpenes, alcohols, etc.). Researchers used electronic nose/tongue with chemometrics for rapid identification of its cultivars and aging years; GC–MS and UHPLC-Q-TOF-MS identified key volatiles including β-myrcene and limonene [3]. Citri Reticulatae Pericarpium (CRP), a widely recognized medicinal and edible homologous herb, has garnered considerable research interest due to its diverse biological properties, including hypolipidemic, anti-inflammatory, antidepressant, and antihypertensive effects [4,5,6], with high value in functional foods and daily chemical industries. Its quality and market value largely depend on characteristic aroma, closely related to processing techniques, making aroma retention crucial for industrial production and societal demand. Current CRP research mainly focuses on active components and pharmacology, while the impact of drying on aroma remains underexplored. As a critical processing step, inappropriate drying causes volatile oil loss and structural damage [7,8]. Conventional thermal and sun drying destroys cell structures, leading to shrinkage and accelerated aroma degradation, impairing CRP’s quality and commercial value. Flavonoids and volatile oils are key CRP quality markers [9], and hot-air drying is superior to sun drying in retaining them [10]. However, existing studies lack systematic characterization combining aroma, sensory properties, and microstructure, leaving a research gap.

Nevertheless, the impact of different drying methods on CRP aroma profiles and related sensory evaluations has been rarely reported. This study selected five-year-stored CRP with different drying methods, integrating GC–IMS, GC–MS, sensory evaluation, electronic nose, and SEM for analysis; PLSR explored aroma–sensory correlations. It supplements theoretical understanding and provides a scientific basis for optimizing drying technologies and selecting high-quality CRP raw materials, supporting the efficient development of the CRP industry. This study provides a scientific basis for sample selection in CRP drying processes and CRP essential oil extraction.

## 2. Materials and Methods

### 2.1. Samples and Chemicals

Citrus Reticulata Peel (CRP) samples subjected to different drying and storage conditions—designated as XH, A, B, and C—were all aged for five years. The control sample (XHCRP) was dried under controlled temperature and humidity conditions and stored in a standard warehouse. Sample A was naturally sun-dried and subsequently stored in stacked piles, while sample B was naturally sun-dried and then stored in a standard warehouse. Sample C was dried using hot-air treatment and stored indoors. This study conducted a comparative analysis of commercially processed products rather than a controlled experiment, acknowledging that their proprietary processing parameters constitute a methodological limitation. All samples were purchased from Xinhui Village, Jiangmen City, Guangdong Province, China. Reference standards, including 2-methyl-3-heptanone (GR), n-alkanes (C7–C30) (GR), and n-hexane (GR), were obtained from Sigma-Aldrich (St. Louis, MO, USA).

### 2.2. Solid-Phase Microextraction (SPME)

A 3 g portion of each sample, along with 1 μL of the internal standard 2-methyl-3-heptanone (analytical standard, ≥99.5%; Sigma-Aldrich, St. Louis, MO, USA) was diluted with dichloromethane (HPLC-grade, ≥99.9%; Sigma-Aldrich, USA) to a final concentration of 0.816 μg/μL and was placed into a 20 mL sealed vial. The vial was equilibrated in a water bath at 55 °C for 20 min. a CAR/DVB/PDMS-coated SPME fiber (65 μm; Supelco Inc., Bellefonte, PA, USA, a subsidiary of Merck KGaA, Darmstadt, Germany)was used and exposed to the headspace for 40 min to adsorb volatile compounds. The fiber was subsequently placed into the gas chromatograph (GC) injection port, where thermal desorption was carried out at 250 °C for 5 min with a split ratio of 5:1 [11].

### 2.3. Analysis of Aroma Compounds in the Samples by GC–IMS and GC–MS

GC–IMS analysis was carried out with a FlavourSpec^®^ instrument (G.A.S, Dortmund, Germany). GC–IMS operating conditions: An MXT-5 capillary column (15 m × 0.53 mm ID) was employed, and the column temperature was maintained at 40 °C. The total analysis time was 32 min. High-purity nitrogen (N_2_, ≥99.999%) was used as the carrier gas, and the IMS drift tube temperature was set to 45 °C. The carrier gas flow rate was initially set at 2.00 mL/min and held for 2 min, then increased linearly to 100.00 mL/min over 0–30 min, after which this flow rate was maintained for an additional 10 min [11].

A GC–MS system (Shimadzu, QP2010 Ultra, Kyoto, Japan) was applied for qualitative and quantitative analysis. Gas chromatography–olfactometry–mass spectrometry (GC–MS) conditions: For GC analysis, a DB-5 capillary column was used with helium (purity > 99.99%) as the carrier gas at a constant flow rate of 1.2 mL/min and a split ratio of 5:1. The oven temperature program started at 40 °C and was held for 3 min, followed by an increase to 200 °C at a rate of 5 °C/min (without holding), and then ramped to 230 °C at 10 °C/min with a final hold of 3 min. MS analysis was performed using electron impact (EI) ionization at 70 eV. The ion source temperature was set to 230 °C, the mass scan range was 40–300 m/z, and the solvent delay time was 4 min [12].

Qualitative and quantitative analysis of aroma compounds: For qualitative identification, the mass spectra of volatile aroma compounds were initially matched against the NIST 2.0 mass spectral library. Retention index (RI) values were then verified using standard odor descriptions, and key aroma compounds were further confirmed based on the odor characteristics of corresponding reference standards. RI values were calculated using a homologous series of n-alkanes according to the following equation:

RI = 100N + 100n (t_Ra_ − t_RN_)/(t_R(N+n)_ − t_RN_) (1)

where N represents the carbon number of the lower n-alkane; n is the carbon number difference between the two bracketing n-alkanes; and t_Ra,_ t_RN_, and t_R(N+n)_ correspond to the retention times of the target aroma compound, the lower n-alkane, and the higher n-alkane, respectively.

Relative quantification was carried out using an internal standard method by comparing the chromatographic peak area of each volatile compound with that of the internal standard. The relative concentration was calculated using the following equation:

C_i_ = C_IS_ × A_i_/A_IS_ (μg/g) (2)

where C_i_ is the relative concentration of the aroma compound, C_IS_ is the relative concentration of the internal standard, A_i_ is the peak area of the aroma compound, and A_IS_ is the peak area of the internal standard. Using an internal standard, semi-quantitative analysis was conducted to initially screen key aroma compounds [13].

### 2.4. Sensory Evaluation

Descriptive sensory analysis: Approximately 15 g of each sample was weighed and transferred into a clean, odor-free 40 mL headspace vial. Sensory evaluation was carried out by a trained panel consisting of 12 assessors (7 females and 5 males, mean age 30 years) in a quiet, odor-controlled sensory laboratory. Panelists were instructed to abstain from eating for at least one hour before the session. To ensure independent assessments, communication among panelists was not permitted during evaluation. Reference samples included five types of authentic CRP and pure chemical standards representing key aroma attributes. Specifically, D-limonene (citrus, 0.1% *v*/*v*), ethyl butanoate (fruity, 0.05% *v*/*v*), linalool (floral, 0.1% *v*/*v*), ethyl butanoate (sweet, 0.05% *v*/*v*), octanal (hay-like, 0.05% *v*/*v*), and citric acid (sour, 0.5% *w*/*v*) were employed as reference standards, following established citrus aroma sensory protocols [14]. Each sample was evaluated in triplicate, with results reported as mean values. The primary aroma descriptors for the citrus peel included fruity, sweet, sour, floral, hay-like, and citrus notes, while refreshing and pleasant characteristics were also assessed using the same 10-point scale [12].

### 2.5. Discrimination of CRP Based on E-Nose Analysis

An E-nose with a gas sensor array (Sensigent, Cyranose 320, Baldwin Park, CA, USA) was used for odor detection. A 3.5 g portion of the CRP sample was transferred into a 50 mL headspace vial, which was subsequently capped, sealed, and equilibrated at 26 °C for 30 min prior to analysis. The electronic nose operating conditions were as follows: cleaning time of 120 s, zeroing time of 5 s, pre-injection time of 8 s, measurement time of 60 s, and a carrier gas flow rate of 400 mL/min. Under these conditions, variations in the conductivity ratio (G/G_0_) were recorded, where G and G_0_ denote the responses of the metal oxide sensor to the sample gas and the clean gas, respectively. All measurements were performed in triplicate, and the mean values were calculated for subsequent plotting and analysis using Origin software. Unlike principal component analysis (PCA), which is primarily used for data reduction, this method focuses on classification by maximizing inter-group separation while minimizing variability within groups.

### 2.6. Scanning Electron Microscope

The CRP microstructures of the four samples were examined using a JSM-6510LV (JEOL, Tokyo, Japan) scanning electron microscope (SEM). For surface analysis, a small section of each CRP was excised from the central region for imaging at magnifications of 200× and 500×. Each specimen was mounted onto the sample stage with conductive adhesive, ensuring a flat orientation to achieve optimal electrical conductivity. Prior to imaging, the samples were coated with a thin layer of gold using an ion sputter coater. Morphological observations were conducted at an accelerating voltage of 20 kV [15].

### 2.7. Statistical Analysis

For GC–MS-derived data, statistical differences among samples were evaluated using analysis of variance (ANOVA) followed by Duncan’s multiple range test. Principal component analysis (PCA) was applied to illustrate sample clustering and to elucidate the contributions of odor-active compounds to overall flavor profiles. GC–IMS data were processed using Laboratory Analytical Viewer (LAV) software (Version 2.7) equipped with three plug-ins, including the GC × IMS Library Search, allowing for multidimensional analysis of samples. Statistical analyses were conducted using IBM SPSS Statistics 22.0 (Chicago, IL, USA). Quantitative results are presented as mean ± standard error of the mean (SEM), with significance set at *p* < 0.05. Heatmaps were constructed using Origin 2019 software. Partial least-squares regression (PLSR) was conducted to correlate volatile compound profiles (X-matrix) with sensory attributes (Y-matrix) of XHCRP. The optimal number of latent variables was determined using leave-one-out cross-validation (LOOCV) to assess the model’s predictive performance and prevent overfitting [16].

## 3. Results and Discussion

### 3.1. Analysis of Volatile Components in CRP Based on GC–IMS

**Figure 1:**
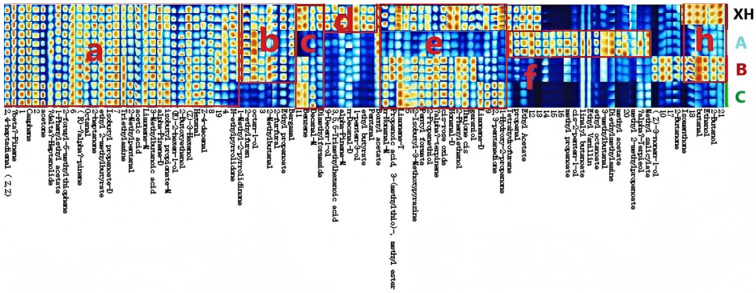
Gallery plot (fingerprint) of CRP samples. (The regions a, b, c, d, e, f, and h in the figure were used to analyze the differences in aroma compounds.)

Significant differences were observed in both the composition and relative abundance of volatile compounds among the four CRP samples. Compounds showing pronounced variation were categorized into seven regions (a, b, c, d, e, f, and h) for detailed discussion (Figure 1). Substances identified in region a were detected across all four dried citrus peel samples and primarily consisted of aldehydes, alcohols, and ketones. Specifically, 2,4-heptadienal (Z,Z), hexanal, and (Z)-3-hexenol contributed strong grassy notes. Limonene and Z-4-decenal imparted characteristic lemon-citrus aromas. β-Pinene exhibited a resinous scent, whereas α-pinene presented a typical pinene odor, and camphene produced a faint camphor-like aroma. δ-Heptanolide was associated with a coconut oil-like note, while 1-phenylethyl acetate contributed a fresh floral aroma. 2-Formyl-5-methylthiophene was characterized by a woody fragrance, and (R)-α-pinene imparted an aromatic, mint-like note. Octanal displayed a pungent, sharp, and waxy aroma. Fruity notes were attributed to 2-heptanone, (E)-2-hexen-1-ol, ethyl 2-methylbutyrate, and isobutyl propanoate. In addition, triethylamine exhibited an ammonia-like odor; 2-methylpentanal imparted a roasted peanut-like aroma; acetic acid was characterized by an irritating odor; 3-methylbutanoic acid exhibited an unpleasant rancid smell; and 2-butoxyethanol contributed a mild aroma.

The compounds identified in region b were commonly found in the XH, A, and B dried citrus peel samples at relatively high concentrations. Among them, 1-ethyl-2-pyrrolidinone had a slight ammonia-like odor; 2-ethylfuran exhibited a roasted aroma; octan-1-ol carried a citrus note; 2-ethylbutanal produced an irritating smell; 2-furfural had a distinctive benzaldehyde-like scent; and ethyl propanoate and Bergamal contributed fruity flavors.

The compounds detected in region c were prevalent in the XH, B, and C dried citrus peel samples with relatively high levels. Benzene exhibited an aromatic odor, while decanal emitted a sweet fragrance reminiscent of orange and lemon.

The compounds in region d were unique to the XH samples and present at relatively high concentrations. Dimethylformamide had an ammonia-like odor; 9-decen-1-ol gave a floral aroma; α-pinene exhibited its characteristic pine scent; Decanal was characterized by a sweet fragrance reminiscent of orange and lemon; 1-penten-3-ol and ethyl butyrate offered fruity notes; and pentanal possessed a distinctive aroma. Unlike other drying methods that easily cause the loss of unique volatile compounds [16], the specific drying and aging processes adopted for the XH samples effectively retained these characteristic aroma substances, resulting in their uniqueness in region d.

The compounds identified in region e were detected in the XH, B, and C samples at relatively high levels, predominantly consisting of esters, aldehydes, and ketones. Specifically, bornyl acetate imparted a cool pinewood aroma; propanoic acid, 3-(methylthio)-, methyl ester and pentyl formate contributed fruity notes; limonene, α-terpinene, and nonanal provided citrusy, lemon-like scents; 2-isobutyl-3-ethoxypyrazine had a green pepper aroma; 2-propanethiol produced an unpleasant odor; cis-rose oxide offered a green floral fragrance reminiscent of rose cortex; 2-phenylethanol and geraniol conveyed floral aromas; and cis-thujone presented a cedar and arborvitae scent [12]. In contrast to tray drying (TD), which was found to cause severe loss of esters and aldehydes in citrus peels [17], the XH, B, and C samples retained significantly higher levels of these aroma-active compounds, suggesting that their drying methods are more superior in preserving the aromatic quality of CRP.

The compounds in region f were detected at relatively high concentrations in both the XH and A samples.Limonene displayed a fresh lemon fragrance, 2,3-pentanedione gave a creamy aroma, and 1-hydroxy-2-propanone emitted a caramel-like scent. Wang et al. [16] demonstrated that drying temperature is a critical factor affecting the content of volatile compounds in citrus peels, and the high abundance of characteristic compounds in the XH and A samples might be associated with their moderate drying temperature.

The compounds in region h were found in the XH and B dried citrus peel samples at relatively high levels. Tetrahydrofuran exhibited an ether-like odor, while propanal had a pungent smell. Ethyl acetate, methyl propanoate, cis-2-penten-1-ol, and methyl 2-ethylpropanoate contributed fruity aromas; linalyl butanoate and 3-ethylbutanal provided a bergamot-like scent; ethyl vanillin offered a vanilla bean fragrance; ethyl octanoate produced a sweet, brandy-like aroma; methyl acetate had a fragrant odor; and α-terpineol emitted a floral scent. Additionally, methyl salicylate conveyed a wintergreen leaf flavor, (Z)-3-nonen-1-ol presented a mushroom-like taste, isomenthone and 2-butanol had minty aromas, and butanal and ethanol emitted sharp, irritating odors.

**Figure 2:**
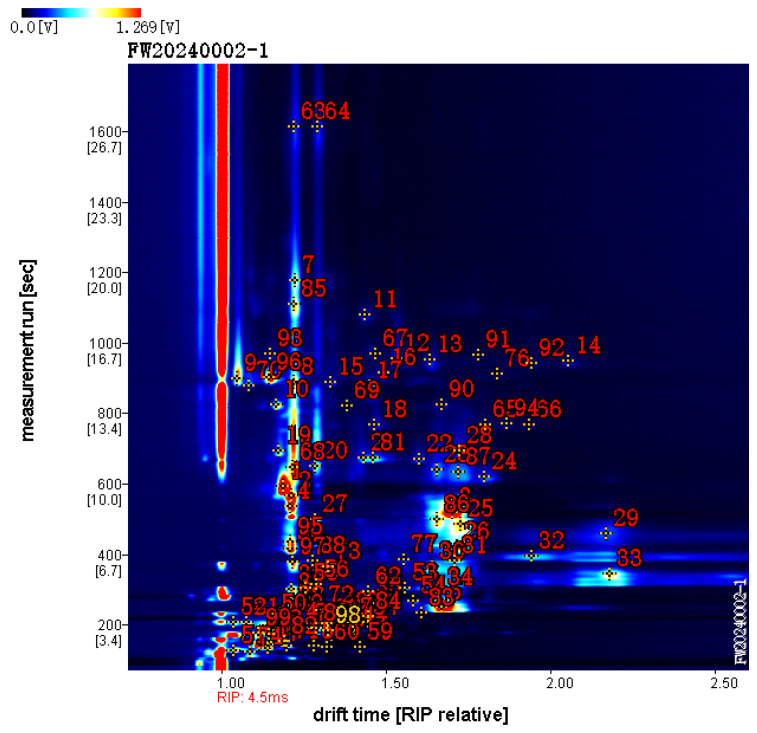
Qualitative analysis of CRP by GC–IMS.

A total of 96 characteristic target volatile compounds were successfully identified from the topographic plots using the GC × IMS Library Figure 2 (Table 1). Compared with the volatile profile of fresh CRP, the drying and aging processes led to the formation of a substantial number of new volatile compounds. Overall, the results shown in Figure 2 indicate that the aroma profile of CRP—primarily composed of terpenes, alcohols, aldehydes, and a minor proportion of furans—undergoes significant alterations depending on the drying method. Its aroma profiles were distinctly different from those of citrus peels dried by vacuum infrared drying (VID) and vacuum microwave drying (VMD). Bozkir et al. [18] reported that VID and VMD can better retain volatile compounds than TD, but the XH and B samples in this study showed a more abundant aroma profile, especially in fruity and floral compounds.

### 3.2. Identification and Quantification of Volatile Components by GC–MS

**Figure 3:**
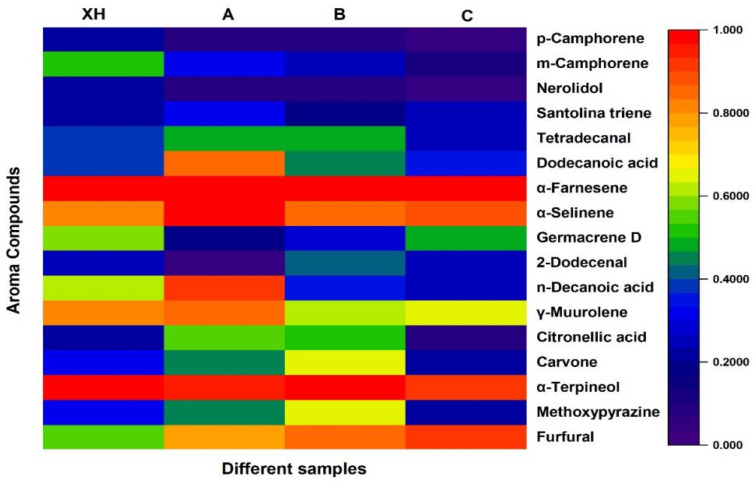
Determination of volatile components by GC–MS.

The primary compounds were identified using the SPME/GC–MS internal standard method, and their corresponding aroma profiles were obtained from the Flavornet database. Differences among the various samples were then analyzed (Figure 3). The results revealed significant variations in the aroma components of CRP subjected to different drying methods, with both the types and concentrations of compounds changing noticeably. As illustrated in the figure, the XH samples generally exhibited higher concentrations of several key compounds, including α-terpineol (floral and woody), γ-muurolene (woody and spicy), 2-dodecenal (sweet), germacrene (woody and spicy), β-selinenol (woody and green), α-farnesene (woody), nerolidol, and m-camphorene. Compared with samples A, B, and C, Xinhui tangerine peel contained a greater diversity of volatile compounds, predominantly esters, alcohols, ketones, aldehydes, and terpenoids, with smaller amounts of acids and heterocyclic compounds. XHCRP was characterized by prominent citrus, sweet fruity, and floral aromas, along with cool notes reminiscent of mint and pine. The broader range of volatile compounds (esters, alcohols, ketones, aldehydes, terpenoids, acids, and heterocyclics) in XHCRP contributes to these complex aroma profiles, enhancing its market quality. Consequently, XHCRP surpasses the other samples in both flavor and overall quality, exhibiting unique aroma characteristics with strong commercial potential [19]. Consistent with this finding, Zheng et al. also verified that the drying regime is a critical factor regulating the bioactive and volatile components in Citri Reticulatae Pericarpium, which directly determines its sensory quality and market value [20].

Specifically, during the drying phase, the dynamic evolution of flavor metabolites, including the synthesis of new aroma compounds and the conversion of their precursor substances, is closely associated with the changes in microbial diversity induced by different drying conditions, which further regulates the formation of CRP aroma profiles [8]. Different drying techniques affect the formation of aroma compounds by altering the drying rate and physical structure of CRP, and they can significantly influence the content of total volatile oil and the composition of volatile organic compounds (VOCs), with thermal drying and non-thermal drying methods leading to distinct differences in aroma-related components [21]. Additionally, the oil cell number, which is affected by drying techniques, can indirectly regulate the accumulation and release of key aroma substances, further participating in the formation of the unique aroma of XHCRP [21].

### 3.3. Sensory Evaluation and Emotion Evaluation

**Figure 4:**
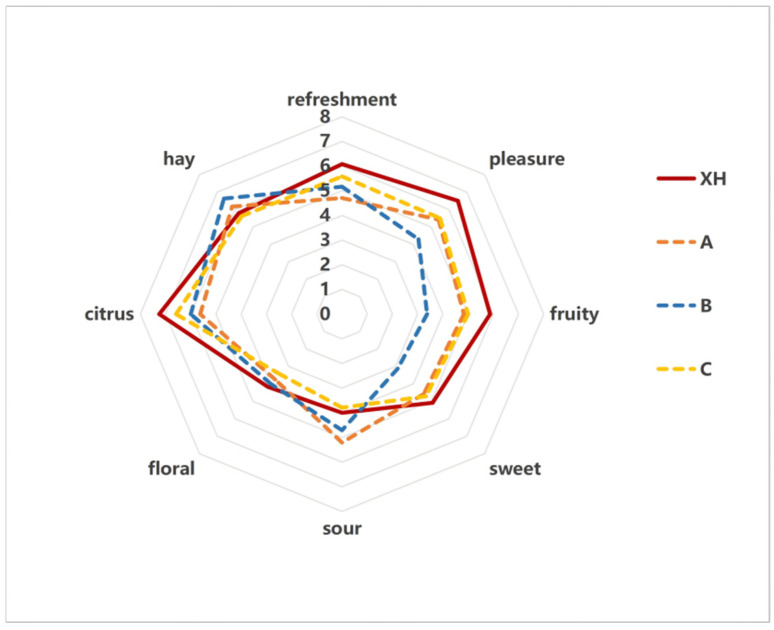
Radar chart of sensory evaluation of different CRP samples.

The radar chart illustrating the aroma quality of CRP from different samples (Figure 4) shows that the overall characteristic aroma sub-attributes included fruity, sweet, sour, floral, hay-like, and citrus notes. A 10-point scale was used to evaluate the refreshing and pleasant sensations associated with the aromas. Sensory evaluation results indicated that the XH sample and sample C were primarily characterized by fruity, citrus, and sweet aromas. This is likely because both samples underwent indoor drying followed by indoor storage, which helped preserve a higher concentration of volatile compounds compared to the other samples. Samples A and B displayed more pronounced hay-like and citrus aromas. Regarding aroma pleasantness and refreshing quality, the XH, C, and A samples received higher sensory scores. Overall, the XH sample showed a distinct advantage in aroma-related sensory evaluation [22].

### 3.4. Electronic Nose Analysis

**Figure 5:**
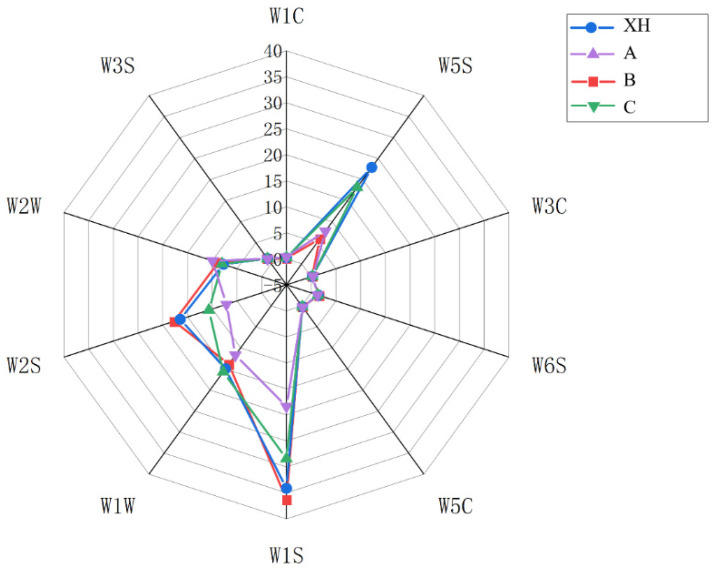
Electronic nose radar map of CRP.

To investigate the response patterns of the 10 sensors in the electronic nose toward CRP samples under different storage conditions, the sensor signals were analyzed to determine each sensor’s response value. The sensor response values were evenly arranged along the circumference of the plot, and the maximum response of each sensor was extracted to generate the radar chart shown in Figure 5. This analysis revealed that the CRP samples contained a diverse array of odor-active compounds. Among the five CRP samples with different storage conditions, sensors W5S (sensitive to nitrogen oxides), W1S (methyl groups), and W1W (sulfides) exhibited the highest response intensities. Sensors W2W (aromatic compounds and organic sulfides) and W2S (alcohols, aldehydes, and ketones) showed moderate responsiveness, while W6S (hydrides), W1C (benzene), W5C (short-chain alkanes), and W3C (aromatic ammonia) displayed the lowest sensitivity. Regarding the samples, XH demonstrated the strongest overall aroma intensity, followed by C and B, with A showing the weakest aromatic profile.

**Figure 6:**
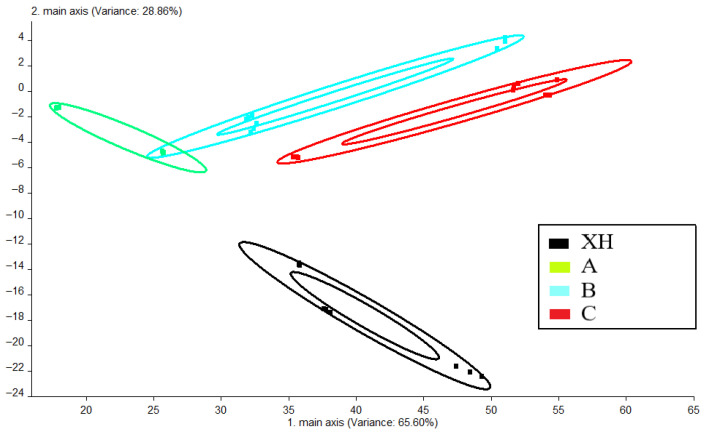
PCA of CRP samples.

As shown in the PCA plot (Figure 6), XHCRP is positioned in the lower region, distinctly separated from the other samples, indicating its unique flavor profile and low similarity to the others. In contrast, samples A, B, and C cluster closely together, reflecting their high flavor similarity.

### 3.5. Scanning Electron Microscopy and Microstructure Analysis of CRP

**Figure 7:**
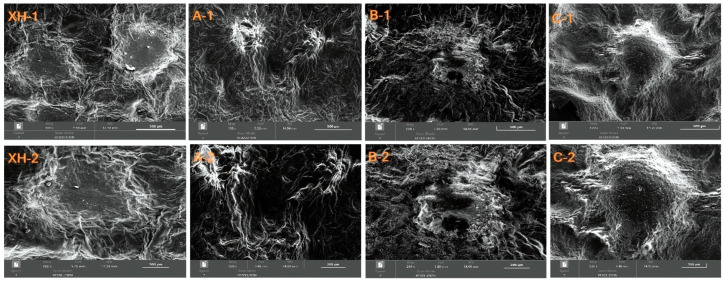
Scanning electron microscope (SEM) microstructure of CRP.

Figure 7 presents the surface morphology of CRP samples from different vintages at magnifications of 200 μm (XH-1, A-1, B-1, C-1) and 500 μm (XH-2, A-1, B-2, C-2). As the peel ages, pericarp cells deteriorate rapidly, and plastids and vacuoles gradually disintegrate, accompanied by respiration and other physiological processes [23]. The pericarp tissue shrinks, its initially loose structure collapses and becomes scattered, and small, sporadically distributed pores appear on the surface. Regarding stomata, the surfaces of samples XH and C, despite exhibiting numerous wrinkles, retain some fullness and three-dimensionality; their stomata collapse into concave shapes with pore sizes larger than those of samples A and B. In sample B (naturally sun-dried), surface wrinkles are more pronounced, shrinkage is greater, and stomatal pores decrease in size. The stomata of the stored XH sample are concave with raised areas surrounding them, whereas those of sample C enlarge significantly, showing a concave center with raised surrounding areas.

These structural variations could be partially explained by the decomposition of non-volatile components in the pericarp during long-term aging, which can be transformed into aroma-contributing small molecules and thus lead to modifications in tissue structure and stomatal morphology [20]. Meanwhile, enzymatic browning also plays an important role: under aerobic conditions, enzymes react with corresponding substrates and catalyze the formation of quinones, which further polymerize or combine with amino compounds to generate brown pigments, resulting in pericarp browning [14,24]. During such processes, the pericarp tissue gradually softens, stomatal structures become indistinct, and the overall tissue becomes looser, which is consistent with previous observations in stored CRP [20].

### 3.6. Correlation Between the Content of Aroma Compounds and Odor During the Storage Period Analyzed by PLSR

**Figure 8:**
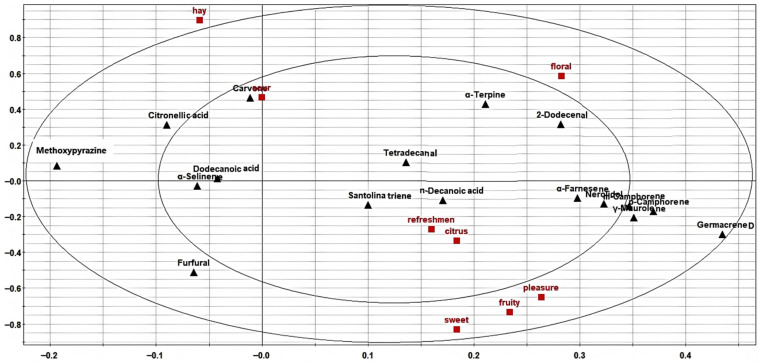
Correlation of aroma compounds and odor by PLSR.

Figure 8 depicts the relationship between various aroma compounds and the odor attributes of the XH CRP sample, as determined by a PLSR model. The upper-left quadrant of Figure 8 highlights significant correlations (*p* < 0.05) between the aroma compounds and specific odor characteristics. Sour and hay-like odors were positively associated with compounds such as methoxypyrazine, D-(+)-carvone, decanoic acid, and santolina triene. Conversely, compounds in the upper-right quadrant, including α-terpineol, tetradecanal, and 2-dodecenal, exhibited negative correlations with these two odor attributes. In the lower-right quadrant, aroma compounds like germacrene, β-selinenol, α-farnesene, santolina triene, nerolidol, and furfural showed positive correlations with fresh, citrus, pleasant, fruity, and sweet odors. Meanwhile, compounds in the lower-left quadrant displayed negative correlations with these odor attributes.

## 4. Conclusions

This study comprehensively analyzed the aroma profiles, flavor variations, surface microstructure, and aroma–sensory relationships of CRP from different sources, processing methods, and vintages, using integrated techniques including SPME/GC–MS, sensory evaluation, electronic nose detection, and multivariate models (PCA and PLSR). Drying methods affected CRP volatile components: indoor-dried and stored XHCRP had a broader volatile spectrum and higher concentrations of key aroma compounds (e.g., α-terpineol and γ-muurolene), with distinctive aromas. Sensory evaluation and electronic nose confirmed XH’s superior aroma quality; PCA distinguished XH from other samples, which clustered closely due to similar flavors. Additionally, CRP microstructure changed notably with aging, showing pericarp cell deterioration, structural collapse, and varied stomatal morphology, mainly driven by non-volatile decomposition and enzymatic browning. It should be noted that the observed differences in the samples were influenced by the combined effects of different drying methods and five-year natural aging. Therefore, the variations in flavor components and sensory characteristics cannot be attributed solely to drying processes but rather to the integrated effects of drying treatment and long-term storage.

This study innovatively integrates multiple analytical techniques to clarify the synergistic effects of sources, processing methods, and vintages on CRP quality, while establishing the relationship between aroma compounds and sensory attributes—filling the research gap of insufficient systematic characterization of CRP from multi-dimensional perspectives. Practically, it provides a solid scientific basis for the selection of raw materials in relevant application fields: guiding the development of safe, clean, and hygienic drying methods for CRP production, and offering theoretical support for the targeted production of CRP-derived products with characteristic aromas, including essential oils, incense, and functional perfumes. This research further promotes the standardized and high-quality production of CRP and expands its application value in related industries, bringing practical benefits to production practices and industrial upgrading.

## Figures and Tables

**Table 1:** Aroma compounds of CRP identified by HS-GC-IMS.

No	Compounds	CAS	Formula	MW	RI	Rt [Sec]	Dt [a.u.]
1	2,4-Heptadienal (Z,Z)	5910-85-0	C_7_H_10_O	110.2	992.3	594.363	1.19059
2	β-Pinene	127-91-3	C_10_H_16_	136.2	977.9	566.167	1.21609
3	**1**	unidentified	*	0	951.8	518.266	1.70461
4	Camphene	79-92-5	C_10_H_16_	136.2	962	536.412	1.21286
5	**2**	unidentified	*	0	723.4	252.033	1.67446
6	Acetone	67-64-1	C_3_H_6_O	58.1	495.2	146.309	1.11905
7	Bornyl acetate	76-49-3	C_12_H_20_O_2_	196.3	1278.1	1177.504	1.2262
8	δ-Heptanolide	3301-90-4	C_7_H_12_O_2_	128.2	1159.2	887.495	1.22255
9	1-Phenylethyl acetate	93-92-5	C_10_H_12_O_2_	164.2	1164.5	898.869	1.05253
10	2-Formyl-5-methylthiophene	13679-70-4	C_6_H_6_OS	126.2	1128.1	824.217	1.17103
11	9-Decen-1-ol	13019-22-2	C_10_H_20_O	156.3	1241.8	1079.988	1.43873
12	Decanal-M	112-31-2	C_10_H_20_O	156.3	1190.7	956.519	1.53577
13	**3**	unidentified	*	0	1189	952.599	1.63795
14	n-Decanal-D	112-31-2	C_10_H_20_O	156.3	1187.9	950.307	2.05673
15	Isomenthone	491-07-6	C_10_H_18_O	154.3	1160.2	889.571	1.33675
16	**4**	unidentified	*	0	1171.7	914.221	1.49666
17	3,5,5-Trimethylhexanoic acid	3302-10-1	C_9_H_18_O_2_	158.2	1155.6	880	1.45377
18	n-Nonanal-M	124-19-6	C_9_H_18_O	142.2	1098.3	767.915	1.47012
19	Bergamal	106-72-9	C_9_H_16_O	140.2	1054.8	692.577	1.17614
20	Limonene-D	138-86-3	C_10_H_16_	136.2	1028.6	650.698	1.28874
21	1-Methyl-2-pyrrolidinone	872-50-4	C_5_H_9_NO	99.1	1043.4	674.026	1.4377
22	Propanoic acid, 3-(methylthio)-, methyl ester	13532-18-8	C_5_H_10_O_2_S	134.2	1042	671.786	1.60356
23	Limonene-T	138-86-3	C_10_H_16_	136.2	1022.6	641.537	1.65772
24	Octanal	124-13-0	C_8_H_16_O	128.2	1007.7	619.164	1.8037
25	α-Pinene	7785-70-8	C_10_H_16_	136.2	932.4	485.322	1.73124
26	2-Heptanone	110-43-0	C_7_H_14_O	114.2	891	422.145	1.72079
27	α-Pinene-M	80-56-8	C_10_H_16_	136.2	941.5	500.568	1.28888
28	**5**	unidentified	*	0	1054.8	692.542	1.72524
29	**6**	unidentified	*	0	917	460.665	2.17224
30	Ethyl 2-methylbutyrate	7452-79-1	C_7_H_14_O_2_	130.2	843.6	366.191	1.6433
31	Isobutyl propanoate-D	540-42-1	C_7_H_14_O_2_	130.2	858.4	382.786	1.71212
32	**7**	unidentified	*	0	869.3	395.468	1.94579
33	**8**	unidentified	*	0	824.3	345.527	2.18164
34	Pentyl formate	638-49-3	C_6_H_12_O_2_	116.2	767.5	290.448	1.66879
35	Ethyl propanoate	105-37-3	C_5_H_10_O_2_	102.1	739.2	265.196	1.4493
36	Triethylamine	121-44-8	C_6_H_15_N	101.2	780.2	302.466	1.21725
37	2-Methylpentanal	123-15-9	C_6_H_12_O	100.2	755.4	279.37	1.21857
38	**9**	unidentified	*	0	707.3	239.372	1.21721
39	Pentanal	110-62-3	C_5_H_10_O	86.1	688.2	225.179	1.42501
40	2,3-Pentanedione	600-14-6	C_5_H_8_O_2_	100.1	675.4	218.86	1.21358
41	Benzene	71-43-2	C_6_H_6_	78.1	648.7	206.176	1.08117
42	Acetic acid	64-19-7	C_2_H_4_O_2_	60.1	627.3	196.55	1.14892
43	2-Butanone	78-93-3	C_4_H_8_O	72.1	591.6	181.459	1.24433
44	**10**	unidentified	*	0	600.5	185.105	1.41118
45	Tetrahydrofurane	109-99-9	C_4_H_8_O	72.1	623.4	194.865	1.23708
46	Propanal	123-38-6	C_3_H_6_O	58.1	509.8	151.175	1.15704
47	**11**	unidentified	*	0	459.7	135.148	1.14505
48	Ethyl acetate	141-78-6	C_4_H_8_O_2_	88.1	564.2	170.712	1.33808
49	1-Penten-3-ol	616-25-1	C_5_H_10_O	86.1	685.5	223.861	1.35015
50	2-Methylbutanal	96-17-3	C_5_H_10_O	86.1	674.2	218.256	1.16324
51	Ethanol	64-17-5	C_2_H_6_O	46.1	435.2	127.946	1.04191
52	1-Hydroxy-2-propanone	116-09-6	C_3_H_6_O_2_	74.1	650.9	207.195	1.04046
53	Ethyl butyrate	105-54-4	C_6_H_12_O_2_	116.2	783.4	305.614	1.56114
54	**12**	unidentified	*	0	740.5	266.306	1.58684
55	Dimethylformamide	68-12-2	C_3_H_7_NO	73.1	783.4	305.64	1.26047
56	Hexanal	66-25-1	C_6_H_12_O	100.2	792	313.653	1.29176
57	**13**	unidentified	*	0	480.3	141.51	1.28705
58	Cis-2-penten-1-ol	1576-95-0	C_5_H_10_O	86.1	771.3	293.941	1.44934
59	Linalyl butanoate	78-36-4	C_14_H_24_O_2_	224.3	1411	1614.95	1.22539
60	Ethyl vanillin	121-32-4	C_9_H_10_O_3_	166.2	1410.9	1614.415	1.29612
61	Cis-rose oxide	3033-23-6	C_10_H_18_O	154.3	1098.4	768.11	1.80756
62	Nonanal-D	124-19-6	C_9_H_18_O	142.2	1098	767.429	1.93877
63	Limonene-M	138-86-3	C_10_H_16_	136.2	1025.7	646.169	1.219
64	(Z)-3-nonen-1-ol	10340-23-5	C_9_H_18_O	142.2	1126.1	820.319	1.38669
65	**14**	unidentified	*	0	1155.3	879.303	1.08637
66	**15**	unidentified	*	0	473	139.235	1.20457
67	2-Ethylfuran	3208-16-0	C_6_H_8_O	96.1	707.2	239.293	1.30514
68	2-Furfural	98-01-1	C_5_H_4_O_2_	96.1	843.9	366.528	1.32698
69	**16**	unidentified	*	0	418.7	123.311	1.09023
70	3-Methylbutanal	590-86-3	C_5_H_10_O	86.1	649.4	206.481	1.40303
71	4-Ketoisophorone	1125-21-9	C_9_H_12_O_2_	152.2	1132.4	832.856	1.33188
72	**17**	unidentified	*	0	1140.2	848.361	1.78974
73	**18**	unidentified	*	0	1160	889.197	1.59659
74	2-Isobutyl-3-methoxypyrazine	24683-00-9	C_9_H_14_N_2_O	166.2	1170.7	912.184	1.84124
75	(E)-2-hexen-1-ol	928-95-0	C_6_H_12_O	100.2	859.5	384.065	1.55833
76	2-Propanethiol	75-33-2	C_3_H_8_S	76.2	569.2	172.629	1.14739
77	3-Methylbutanoic acid	503-74-2	C_5_H_10_O_2_	102.1	886.5	416.454	1.21233
78	Octan-1-ol	111-87-5	C_8_H_18_O	130.2	1042.9	673.228	1.46523
79	Methyl acetate	79-20-9	C_3_H_6_O_2_	74.1	518.3	154.058	1.19534
80	**19**	unidentified	*	0	700.9	234.457	1.61102
81	Methyl 2-methylpropanoate	547-63-7	C_5_H_10_O_2_	102.1	683.3	222.734	1.45116
82	Geraniol	106-24-1	C_10_H_18_O	154.3	1253.4	1110.237	1.22551
83	Alpha-pinene-D	80-56-8	C_10_H_16_	136.2	939.9	497.835	1.65887
84	α-Terpinene	99-86-5	C_10_H_16_	136.2	1017.8	634.162	1.72181
85	Isobutyl propionate-M	540-42-1	C_7_H_14_O_2_	130.2	858.4	382.774	1.28135
86	Butanal	123-72-8	C_4_H_8_O	72.1	612.1	189.978	1.29234
87	2-Phenylethanol	60-12-8	C_8_H_10_O	122.2	1127.6	823.422	1.67124
88	Z-4-decenal	21662-09-9	C_10_H_18_O	154.3	1184.9	943.394	1.94593
89	Methyl salicylate	119-36-8	C_8_H_8_O_3_	152.1	1196.8	970.594	1.15109
90	**20**	unidentified	*	0	1146.2	860.634	1.24883
91	Thujone cis	546-80-5	C_10_H_16_O	152.2	1099.7	770.466	1.87156
92	2-Butoxyethanol	111-76-2	C_6_H_14_O_2_	118.2	900.2	435.207	1.21228
93	N-ethylpyrrolidone	2687-91-4	C_6_H_11_NO	113.2	1165.9	901.886	1.15055
94	(Z)-3-hexenol	928-96-1	C_6_H_12_O	100.2	853.9	377.686	1.21998
95	2-Butanol	78-92-2	C_4_H_10_O	74.1	597.5	183.888	1.3279
96	**21**	unidentified	*	0	582	177.606	1.11674

“*” Not identified in the standard library; chemical formula unknown.

## Data Availability

The original contributions of this study are presented in this article. Further inquiries can be directed to the corresponding author.

## References

[1] Zhang H., Cui J., Tian G., DiMarco-Crook C., Gao W., Zhao C., Li G., Lian Y., Xiao H., Zheng J. (2019). Efficiency of four different dietary preparation methods in extracting functional compounds from dried tangerine peel. Food Chem..

[2] Fu M., Wang Y., Yu Y., Wen J., Cheong M.S., Cheang W.S., Wu J. (2022). Changes of volatile substance composition during processing of nine-processed tangerine peel (Jiuzhi Chenpi) determined by gas chromatography-ion mobility spectrometry. Front. Nutr..

[3] Li X., Yang Y., Zhu Y., Ben A., Qi J. (2022). A novel strategy for discriminating different cultivation and screening odor and taste flavor compounds in Xinhui tangerine peel using E-nose, E-tongue, and chemometrics. Food Chem..

[4] Yu Z., Wu Y., Ma Y., Cheng Y., Song G., Zhang F. (2022). Systematic analysis of the mechanism of aged citrus peel (Chenpi) in oral squamous cell carcinoma treatment via network pharmacology, molecular docking and experimental validation. J. Funct. Foods.

[5] Zou J., Wang J., Ye W., Lu J., Li C., Zhang D., Liu Z. (2022). Citri Reticulatae Pericarpium (Chenpi): A multi-efficacy pericarp in treating cardiovascular diseases. Biomed. Pharmacother..

[6] Wang J., Hao J., Miao D., Xiao P., Jiang X., Hu L. (2024). Compound chenpi tea consumption reduces obesity-related metabolic disorders by modulating gut microbiota and serum metabolites in mice. J. Sci. Food Agric..

[7] Talens C., Arboleya J., Castro-Giraldez M., Fito P. (2017). Effect of microwave power coupled with hot air drying on process efficiency and physico-chemical properties of a new dietary fibre ingredient obtained from orange peel. LWT.

[8] Sun W., Li M.J., Zhang Y., Ai Z.P., Lei D.W., Pei Y.P., Liu Y.H. (2023). Effect of different drying techniques on drying characteristics, physical quality, and active components of *Citri reticulatae pericarpium*, and the correlation between physiochemical quality. Ind. Crops Prod..

[9] Li D., Mujumdar A.S., Wen Y. (2020). Hot air impingement drying kinetics and quality attributes of orange peel. J. Food Process. Preserv..

[10] Zheng H., Su Z.C., Huang S.T., Li D.L., Yuan Z.D., Xu J.C. (2025). 4-vinylguaiacol in citri reticulatae ‘chachiensis’ pericarpium volatile oil: A microbial-mediated aging marker enhances glucose metabolism. Foods.

[11] Sireyil G., Alim A. (2022). Effects of onion paste on flavor of a different kind of bread (naan) analyzed with E-Nose and GC–IMS. J. Food Process. Preserv..

[12] Alim A., Huang C.F., Zhao X., Sarengaowa, Zhang R.R., Zhang J.R., Zhang X.Q., Jin Y.B., Wu G.R., Hu W.Z. (2025). GC–IMS/GC–MS/E-nose and molecular simulation analysis of the effects of aging duration on the aroma of Citri Reticulatae Pericarpium and its associated antidepressant effect. J. Agric. Food Res..

[13] Alim A., Song H., Liu Y., Zou T., Zhang Y., Zhang S., Raza A. (2019). Research of beef-meaty aroma compounds from yeast extract using carbon module labeling (CAMOLA) technique. LWT.

[14] González-Mas M.C., Rambla J.L., López-Gresa M.P., Blázquez M.A., Granell A. (2019). Volatile Compounds in Citrus Essential Oils: A Comprehensive Review. Front. Plant Sci..

[15] Ullah F., Papini A., Shah S.N., Zaman W., Sohail A., Iqbal M. (2019). Seed micromorphology and its taxonomic evidence in subfamily Alsinoideae (Caryophyllaceae). Microsc. Res. Tech..

[16] Dong T., Tian Z., Wang S., Jie S., Hai C., Shu W., Bao S. (2024). Identification of key off-flavor compounds during storage of fried pepper (*Zanthoxylum bungeanum* Maxim.) oils by sensory-directed flavor analysis and partial least squares regression (PLSR). J. Food Compos. Anal..

[17] Wang J., Wang H., Xiao W. (2022). Effects of drying temperature on the drying characteristics and volatile profiles of Citrus reticulata Blanco peels under two stages of maturity. Dry. Technol..

[18] Bozkir H., Yeliz T., Erten S. (2021). Effects of tray drying, vacuum infrared drying, and vacuum microwave drying techniques on quality characteristics and aroma profile of orange peels. J. Food Process Eng..

[19] Qin K., Zheng L., Cai H., Cao G., Lou Y., Lu T., Cai B. (2013). Characterization of chemical composition of Pericarpium Citri reticulatae volatile oil by comprehensive two-dimensional gas chromatography with high-resolution time-of-flight mass spectrometry. Evid.-Based Complement. Altern. Med..

[20] Zheng G., Chao Y., Liu M., Yang Y., Zhang D., Wang K., Tao Y., Zhang J., Li Y., Wei M. (2021). Evaluation of dynamic changes in the bioactive components in Citri Reticulatae Pericarpium (*Citrus reticulata* ‘Chachi’) under different harvesting and drying conditions. J. Sci. Food Agric..

[21] Hu Y., Hu D.L., Yin L., Deng Z., Cheng Y.Y., Li H.X., Wang F., Liu Y.P. (2025). Dynamic profiling of bioactive compounds, flavor metabolites, and quality-related microorganisms during the freshening-drying-aging process of Citri reticulatae pericarpium: Implications for quality formation mechanisms. Food Chem. X.

[22] Yang D., Wu X., Shi H., Zhang J., Wang C. (2022). Essential aroma substances and release pattern of Xin hui Chenpi. Beverage Plant Res..

[23] Xu G., Zhao J.Y., Yao J.Q., Xu Y., Yuan X.H., Pan S.Y. (2025). Effects of aging on the fine structure, chain conformation, and morphology of Chenpi polysaccharides. Carbohydr. Polym..

[24] Yi Z.B., Yu Y., Liang Y.Z., Zeng B. (2008). In vitro antioxidant and antimicrobial activities of the extract of Pericarpium Citri Reticulatae and its main flavonoids. LWT.

